# Heterogeneity of compassion fatigue and its relationship with resilience among nurses: a latent profile analysis

**DOI:** 10.3389/fpubh.2026.1818443

**Published:** 2026-04-13

**Authors:** Qian Yao, Jian Luo, Yu Ni, Hua Jian, Ting Xu, Wei Tian, Baomei Song, Xiuchuan Li, Jimei Zhang

**Affiliations:** 1 Department of Emergency, The Affiliated Hospital of Southwest Jiaotong University, The Third People's Hospital of Chengdu, Chengdu, China; 2 Department of Orthopedics, The Fifth People's Hospital of Sichuan Province, Chengdu, China; 3 School of Nursing, Chengdu Medical College, Chengdu, China; 4 Department of Nursing, Zhongjiang County People's Hospital, Zhongjiang, China; 5 School of Nursing, Chengdu University of Traditional Chinese Medicine, Chengdu, China; 6 Intensive Care Unit, Sichuan Lansheng Brain Hospital, Chengdu, China; 7 Department of Cardiology, The General Hospital of Western Theater Command PLA, Chengdu, China

**Keywords:** compassion fatigue, latent profile analysis, nurse, nurses, resilience

## Abstract

**Background:**

Nurses often face high-intensity emotional load and work pressure in clinical work. This working environment makes nurses prone to compassion fatigue (CF), and resilience plays an important role in alleviating CF.

**Aims:**

The purpose of this study is to identify the latent profile of nurses' CF and its population characteristics through latent profile analysis (LPA), and to explore the impact of resilience on the latent profile of CF.

**Design:**

This study is a cross-sectional study.

**Methods:**

A total of 453 nurses from six hospitals in Sichuan Province, China were recruited from December 2024 to April 2025. The nurses were surveyed using a general information questionnaire, a resilience scale, and a compassion fatigue scale. The latent profile of nurses‘ CF was determined by LPA using Mplus 8.3 software. IBM SPSS Statistics 27.0 software was used to explore the factors affecting the latent profile of nurses' CF through chi-square tests, one-way analysis of variance, and multivariate logistic regression analysis.

**Results:**

There were three latent profiles of nurses‘ CF, namely Low CF (*n* = 203, 44.8%), Medium CF (*n* = 115, 34.2%) and High CF (*n* = 95, 21%), which indicated that most nurses were in low and medium CF. Resilience, the number of daily nursing patients, and the number of night shifts per week may be influencing factors of the potential profile of nurses' CF (*P* < 0.05).

**Conclusion:**

CF of nurses can be divided into three potential profiles, and each potential profile is affected by different factors. Nursing managers should regularly assess and identify nurses with moderate or high levels of CF, rationally formulate scheduling plans and assign work tasks, and take targeted intervention measures to enhance nurses' resilience, which will help improve their quality of professional life.

## 1 Introduction

Healthcare workers refers to those who directly or indirectly provide medical services, support or manage related work in the healthcare system. This group usually includes doctors, nurses, medical assistants, nursing assistants, laboratory technicians, administrative staff, and managers, etc. (1). Due to long-term high-pressure working environments, they not only face heavy workloads, but also often witness the pain and struggles of patients. In addition, complex relationships with colleagues often make them feel isolated and helpless at work. Under the combined effect of these multiple factors, compassion fatigue (CF) is prevalent among healthcare workers (2, 3). Among healthcare workers, nurses have a significantly higher level of CF than other types of healthcare workers (2). Nurses account for about 50% of the global healthcare industry, reflecting the high dependence of medical services on nursing. This is mainly due to the key role played by nurses in medical services. They play an important role in health promotion, disease prevention and provision of medical services (4). Compared with other healthcare workers, nurses not only need to provide continuous bedside care, but also often participate in communication with patients and their families (2, 5) to meet their more care needs (6). Due to long-term close contact with patients, nurses are more sensitive to patients' pain, which makes CF more common among nurses and becomes more serious over time (7).

Several systematic reviews have shown that nurses' CF is the result of a combination of factors, including demographic factors, work-related factors, and physical and mental health factors (8–13). Studies have shown that female nurses are more likely to experience CF (8), nurses with higher education levels are better at coping with CF (9), and nurses who are married (9) or older (8) tend to feel more exhausted due to the dual pressure of balancing family and work. Nurses with longer working years tend to have lower levels of CF due to their rich experience (8). In terms of work-related factors, heavy workloads (10), high nurse-patient ratios (14), and irregular night shifts (8) can significantly increase nurses' psychological stress. In addition, the work environment and organizational culture (11, 12), insufficient support between the unit and colleagues (13), and low salary levels (9) also lead to nurses feeling frustrated and exhausted. Physical and mental health issues also play an important role in CF. Poor physical condition directly affects nurses' work performance (13), while factors such as poor sleep quality (13), anxiety, depression, and psychosocial stress (12) can exacerbate the occurrence of CF. Therefore, nurses' CF is formed by the interweaving of multiple factors.

CF was first proposed by Figley, who described it as the emotional exhaustion and functional impairment caused by long-term exposure to work-related stress and empathy pressure when dealing with traumatized individuals (15). CF consists of two parts: burnout and secondary traumatic stress. Burnout refers to an individual's reaction to long-term exposure to complex interpersonal relationships, characterized by emotional exhaustion, depersonalization, and reduced personal accomplishment (16). Secondary traumatic stress refers to negative emotions and behavioral reactions caused by exposure to others‘ traumatic experiences (17). The resulting CF can manifest as a series of symptoms, such as nightmares, hypervigilance, disrupted sleep patterns, inattention, fatigue, weakness, anger, loneliness, and helplessness (18–20). A systematic review included 28,509 nurses from 11 countries and found that the average scores for burnout and secondary trauma were 26.64 and 25.24, respectively, indicating that the CF problem among nurses worldwide is very serious (7). Wang et al. (21) conducted a survey on 1,044 nurses in China and found that the average scores of nurses' burnout and secondary trauma were 27.36 and 26.88, respectively. This revealed that Chinese nurses also face serious problems with CF. Another study showed that the incidence of burnout and secondary traumatic stress among nurses in the Philippines was as high as 74.38% and 83.47% (22). In particular, in departments such as oncology (9), emergency departments (18), and intensive care units (ICU) (7, 23), the incidence of CF among nurses is higher due to the nature of their work and the particularity of their working environment.

When nurses experience severe CF, it not only affects their mental health but may also have a negative impact on the quality of care and medical experience of patients. Studies have shown that severe CF can increase nurses' psychological stress, leading to anxiety, depression, and suicidal behavior (24, 25). In addition, CF can reduce nurses' job satisfaction (26), causing them to lack work motivation, develop professional burnout, and even increase their willingness to leave, resulting in a large number of nursing staff turnover (27–29). For patients, CF can cause nurses to show indifference or alienation when caring for patients, reducing their nursing ability and affecting the quality of nursing services (30). In addition, CF can also weaken nurses' perception of patient safety and affect patient safety (31). Specifically, it can manifest as the occurrence of medical errors, such as communication barriers, falls, omissions in patient monitoring, and medication errors, which ultimately cause great harm to patients (32). Studies have shown that the prevalence of CF among nurses in different regions varies (33), and nurses in Asia have the most serious CF (7). As the largest country in Asia and the most populous country in the world, China has relatively tight medical resources. As of 2020, the number of registered nurses in China was 4.709 million, with only 3.34 registered nurses per 1,000 people, far lower than the 11.9 in the United States. In addition, China is currently facing a serious problem of an aging population, with an insufficient and unevenly distributed nursing workforce, which further exacerbates the pressure on nursing services (34). In China, nurses generally work long hours, with a common shift system, and night and holiday shifts becoming the norm. This work model results in nurses lacking sufficient rest and recovery time, which seriously affects their mental health (35). At the same time, in the Chinese public's perception, the nursing profession is often regarded as a low-income and low-status job (36). Influenced by the traditional Chinese culture of favoring men over women, male nurses also face gender discrimination and encounter many difficulties in their career development (37). This cultural background makes Chinese nurses feel isolated and helpless in terms of career growth and psychological support, and lack the necessary emotional support. Therefore, it is necessary to gain a deeper understanding of the problem of CF among Chinese nurses in order to provide effective solutions to improve their mental health.

In recent years, there has been an increasing number of studies on resilience and nurses' CF, and nurses' mental health remains a research focus (38). Resilience is a personality trait that refers to an individual's ability to adapt, recover, and grow quickly when faced with adversity and setbacks. This psychological resource helps individuals maintain psychological stability and functional integrity when facing stress (39). Studies have shown that resilience has a negative predictive effect on nurses' CF, which means that resilience may help nurses alleviate the negative impact of CF by enhancing their self-regulation ability (40). Nurses with higher resilience can more effectively cope with stress and traumatic events, maintain an optimistic and positive attitude (41), and effectively reduce anxiety and depression symptoms by enhancing their emotional regulation ability, thereby maintaining a good mental health state (42, 43). In addition, studies have shown that resilience plays a mediating role between CF and nursing quality. Improving nurses' resilience can, to a certain extent, alleviate the negative impact of CF on nurses' nursing service quality and job satisfaction (26). Therefore, resilience is considered an important protective factor against CF (44). Previous scholars have verified this in different groups, including nursing students (45), registered nurses (40), and doctors (46), and have demonstrated the importance of resilience in alleviating CF.

This study was guided by the comprehensive theoretical model of CF. The model states that lack of resources, insufficient positive feedback, and nurses‘ responses to personal suffering are the main factors leading to CF (47). The model emphasizes that each nurse has their own resources, which include object resources (hospital infrastructure and adequate staffing), condition resources (marital status), personal resources (professional skills, leadership, self-esteem, and adaptability), and energy resources (physical/emotional energy and time) (47). When faced with patient needs, nurses will assess their own resource status. If their resources are sufficient, then the patient's needs will not threaten their resources, and nurses will invest various resources to provide compassionate care. At the same time, nurses also hope to receive some positive feedback through this process, such as good self-evaluation, positive patient outcomes, and praise from patients, families, and colleagues. This positive feedback will give patients a sense of compassion satisfaction. However, if nurses invest various resources but do not receive positive feedback, CF will occur (47). Conversely, when nurses' resources are scarce, the patient's needs will threaten their resources. They may ignore the patient and focus on themselves instead. After self-reflection, nurses will experience personal pain, which will lead to aversion to the patient's predicament. When the nurse's personal pain increases, they will also not receive positive feedback from patients, families and colleagues, which will also cause the nurse to experience CF (47). The comprehensive theoretical model of CF provides a good theoretical guidance for our research. The model emphasizes that the lack of resources is one of the main factors leading to nurses‘ CF. In this study, we introduced demographic variables, work-related variables and psychological variables to explore their impact on nurses' CF. These variables can be regarded as resources possessed by nurses, such as object resources (labor relations, number of patients cared for daily, monthly income), conditional resources (gender, age, years of work, and marriage), personal resources (education and resilience) and energy resources (number of night shifts per week and health status). Whether nurses can fully obtain different types of resources and whether they can obtain positive feedback from various levels will be an important factor affecting CF.

Although there is an increasing number of studies on nurses‘ CF (48), most previous studies have evaluated the current status of nurses' CF as a whole and predicted its related influencing factors (49–51). Only a small number of studies have explored the heterogeneity of CF among ICU nurses, and the results showed that ICU nurses were divided into three latent profiles: low, medium, and high (52). Latent profile analysis (LPA) is a method of identifying hidden group structures in data by analyzing continuous data variables. It divides individuals into subgroups with similar characteristics, thereby revealing the heterogeneity of data. This analysis method provides an important reference for achieving precise intervention (53). Therefore, this study aims to identify the heterogeneity of nurses‘ CF through LPA. Guided by the comprehensive theoretical model of CF, our research hypotheses are: (1) there are different latent profiles of nurses' CF; (2) demographic variables or work-related variables will affect the level of nurses‘ CF in different latent profiles; (3) Resilience will affect the level of nurses' CF in different latent profiles. This study aims to identify the heterogeneity of nurses‘ CF and explore the needs and challenges of nurses in different latent profiles in depth. This study provides a reference for nursing managers to develop more precise and personalized intervention measures, thereby effectively reducing nurses' CF and improving their job satisfaction and work motivation.

## 2 Methods

### 2.1 Participants

This study is a cross-sectional study in a descriptive study. We used a convenient sampling method to select 453 nurses from 6 hospitals in Sichuan Province, China. The survey period was from December 2024 to April 2025. Inclusion criteria: (1) having a Chinese nurse qualification certificate; (2) having more than 1 year of clinical work experience; (3) obtaining informed consent and voluntarily participating in this study. Exclusion criteria: (1) intern nurses; (2) nurses in standardized training; (3) nurses who were not working due to further studies or maternity leave.

### 2.2 Sample

This study adopted the cross-sectional sample size calculation formula $n=\frac{Z_{1-a/2}^{2}\sigma^{2}}{d^{2}}$ (54) and set α to 0.05, resulting in Z(1–α/2) of 1.96. The standard deviation σ of the CF score was found to be 24.76 through preliminary survey analysis. The precision d was set to 2.1, resulting in *n* = 272. Considering the dropout rate of 20%, the minimum sample size was determined to be 327 cases.

### 2.3 Data collection

This study used an electronic questionnaire developed using the WJX website (https://www.wjx.cn/). Before the formal survey began, a pilot survey of 30 nurses was conducted. Subsequently, members of the research team met with head nurses from various departments at six hospitals in Sichuan Province, China, detailing the study objectives and precautions. These head nurses then forwarded the questionnaire to eligible nurses in their respective hospitals via WeChat. The first page of the questionnaire contained an informed consent form, which emphasized the anonymity of the questionnaire and explained how to protect patient privacy and data security. In the electronic informed consent form, patients were informed that, to ensure data integrity and validity, each question on the electronic questionnaire was mandatory. Furthermore, to prevent duplicate submissions, each user's IP address could only be entered once. Given that IP addresses may be personally identifiable, the informed consent form explicitly stated that participant responses would not be traced back to individuals and that IP addresses would be deleted during data processing. All researchers also received privacy protection training to enhance their awareness. The collected raw data will be strictly encrypted and stored on a USB drive. The USB drive was physically isolated from the internet and kept solely by the corresponding author. If researchers need to access the data, they must obtain the corresponding author's consent, and data access is limited to personnel involved in this study. The informed consent form also provides contact information for the team, so participants can contact them promptly if they have any questions about the informed consent form. After ensuring that participants fully understand the informed consent form, they can make a voluntary decision. Once they agree to participate, they can choose to withdraw at any time, even during the survey, without submitting the questionnaire.

The survey duration for this study ranged from 3 to 20 min. After the questionnaires were distributed, two researchers reviewed and entered the data. A total of 478 questionnaires were distributed. During the data review process, we eliminated 25 questionnaires, including 11 with strong regularity in the answers, 9 with obvious logical errors, and 5 with a response time of less than 3 min. Ultimately, 453 valid questionnaires were obtained, resulting in a 94.77% response rate.

### 2.4 Measurements

#### 2.4.1 General information questionnaire

After referring to relevant literature, this research team compiled a general information questionnaire on their own, which included 10 items, namely gender, age, education level, marital status, working years, labor relationship, monthly income, number of daily nursing patients, number of night shifts per week, and health status.

#### 2.4.2 Resilience

The Resilience Scale was originally developed by Connor and Davidson (55), and was later translated and revised by Chinese scholars Yu and Zhang (56). The scale consists of three dimensions and 25 items, namely tenacity (13 items), strength (8 items), and optimism (4 items). Each item uses the Likert 5-point scoring method, where 0 represents “never” and 4 represents “almost always.” The total score is 0–100 points, and the higher the score, the better the resilience. The Cronbach's α of the scale is 0.91. In this study, the Cronbach's α was 0.969, and the reliability is good.

#### 2.4.3 Compassion fatigue

The compassion fatigue scale was originally developed by Adams (57) and later translated and revised by Chinese scholar Lou. This study used the Chinese version of the scale (58). The scale consists of two dimensions and 13 items, namely secondary traumatic stress (5 items) and occupational burnout (8 items). Each item uses a 10-point Likert rating method, where 1 represents “never” and 10 represents “very often.” The severity of CF is divided into three levels: low, medium, and high based on the average score of the items, with 1–4 points representing low level, 4–7 points representing medium level, and 7–10 points representing high level. The total score of the scale is 13–130 points, and the higher the score, the more severe the CF. The Cronbach's α of the scale is 0.90. In this study, the Cronbach's α was 0.946, which has good reliability.

### 2.5 Data analysis

In this study, IBM SPSS Statistics 27.0 software was used to analyze the data. The quantitative data were described by means and standard deviations, and the count data were described by frequencies and percentages. Mplus 8.3 software was used to analyze the latent profile model of CF. Model fitting indices included: (1) Information indices: Akaike Information Criterion (AIC), Bayesian Information Criterion (BIC), and adjusted Bayesian Information Criterion (aBIC). The smaller the statistical value, the better the fitting effect (59). (2) Classification indices: When information entropy (Entropy) > 0.8, the classification accuracy reached 90% (60). The closer to 1, the more accurate the classification. (3) Likelihood ratio indices: Lo-Mendell-Rubin (LMR) test and Bootstrap likelihood ratio test (BLRT). When *P* < 0.05, the k-th category model is better than the k-1-th category model (60). Finally, the final number of categories was determined based on the actual significance of the classification. After determining the latent profile of nurses' CF, the χ2 test or one-way ANOVA was used to identify statistically significant variables between different profiles of CF (*P* < 0.05). These statistically significant variables were then included in the multivariate logistic regression analysis to finally determine the factors affecting the latent profile of CF, with the test level of α = 0.05.

### 2.6 Ethics statement

The design of this study strictly follows the principles of the Helsinki Declaration and has been approved by the Ethics Review Committee of The Third People's Hospital of Chengdu before data collection (Number: 2024-S-380). All respondents signed informed consent before the survey.

## 3 Results

### 3.1 Participant characteristics

Among the 453 nurses, most of the participants were female 406 (89.6%), 215 (47.5%) were aged 31–40 years, 346 (76.4%) are undergraduates, 258 (57%) were unmarried, divorced or widowed, 177 (39.1%) had 1–5 years of work experience, 386 (85.2%) were contractors, 345 (76.2%) had a monthly income of 50,000–10,000 CNY, 222 (49%) cared for 1–5 patients on a daily basis, 220 (48.6%) worked night shifts ≤ 1 time per week, and 242 (53.4%) were in sub-healthy health, as shown in Table 4.

### 3.2 Exploratory latent profile analysis

Based on the 13 items of CF, this study conducted a fitting analysis on 1–5 latent profile models. As shown in Table 1, with model 1 as the benchmark, its classification probability is 100%. (1) As the number of models increases, the values of fit indicators such as AIC, BIC, and aBIC gradually decrease, indicating that the model fit has improved. (2) The entropy value reflects the accuracy of classification. The larger the entropy value, the better the classification effect. The entropy value of all models is ranked as model 2> model 5> model 3> model 4, indicating that the classification effect of model 2 is more accurate. (3) The likelihood ratio indicator evaluates the significance of the model. The LMP test *P* value of model 2 and model 3 is < 0.05, while the LMP test *P* value of model 4 and model 5 is >0.05. This shows that model 2 and model 3 are statistically significant, while model 4 and model 5 are not statistically significant. In addition, the sample size of the group in Model 4 is 7.1%, and the sample size of the group in Model 5 is 7.7% (both < 10%), indicating that the total sample size of Model 4 and Model 5 differs significantly between the groups. (4) When the *P* value of the likelihood ratio index LMR test and BLRT test is < 0.05, it means that the k-th category model is better than the k-1-th category model. Based on the above information, we know that only Model 2 and Model 3 are statistically significant (*P* < 0.05). Although the entropy value of Model 2 (0.952) is greater than the entropy value of Model 3 (0.937), the entropy value is not a decisive indicator for determining the number of potential profiles. The AIC, BIC, and aBIC values of Model 3 are all lower than those of Model 2, indicating that the fitting effect of Model 3 is better than that of Model 2. Finally, considering the fitting indices of each model and the practical significance of the classification, we selected Model 3 as the best fitting model.

**Table 1:** Latent profile fitting information of nurses' CF (*N* = 453).

Classes	AIC	BIC	aBIC	Entropy	LMR	BLRT	Categorical probability (%)
1	27,160.112	27,267.126	27,184.611	—	—	—	—
2	24,193.204	24,357.840	24,230.894	0.952	< 0.001	< 0.001	0.567/0.433
**3**	**23,351.815**	**23,574.073**	**23,402.696**	**0.937**	**0.0095**	**< 0.001**	**0.448/0.342/0.210**
4	22,982.074	23,261.955	23,046.146	0.934	0.2657	< 0.001	0.426/0.241/0.263/0.071
5	22,696.830	23,034.333	22,774.094	0.941	0.2651	< 0.001	0.402/0.223/0.075/0.223/0.077

Bold values indicate the optimal model; Abbreviations: AIC, Akaike Information Criterion; BIC, Bayesian Information Criterion; aBIC, adjusted BIC; LMR, Lo-Mendell-Rubin Test; BLRT, Bootstrap Likelihood Ratio Test.

To compare the differences in CF among the three latent profiles, we conducted a one-way analysis of variance. As shown in Table 2, there were significant differences in the scores of CF and its two dimensions across the three latent profiles (P < 0.05). Because the scores of CF and its two dimensions were normally distributed and did not meet the homogeneity of variance test, we conducted *post hoc* pairwise comparisons using Tamhane's T2 test. The results of the Tamhane's T2 test showed that Profile 1 had the lowest CF score, while Profile 3 had the highest CF score.

**Table 2:** Latent profile scores of nurses' CF.

Variables	Total sample (*N* = 453) M ±SD	Profile 1 (*N* = 203) Low CF	Profile 2 (*N* = 115) Medium CF	Profile 3 (*N* = 95) High CF	F	*P*	Tamhane's T2
Secondary traumatic stress	14.79 ± 9.66	6.99 ± 2.73	16.16 ± 5.31	29.24 ± 6.36	757.960	< 0.001	1 < 2 < 3
Burnout	30.26 ± 16.26	15.13 ± 5.70	37.29 ± 7.84	51.14 ± 9.19	897.563	< 0.001	1 < 2 < 3
Total	45.06 ± 24.78	22.11 ± 7.47	53.45 ± 9.39	80.38 ± 13.24	1,286.747	< 0.001	1 < 2 < 3

M, Mean, SD, standard deviation, F, One-way ANOVA.

**Figure 1:**
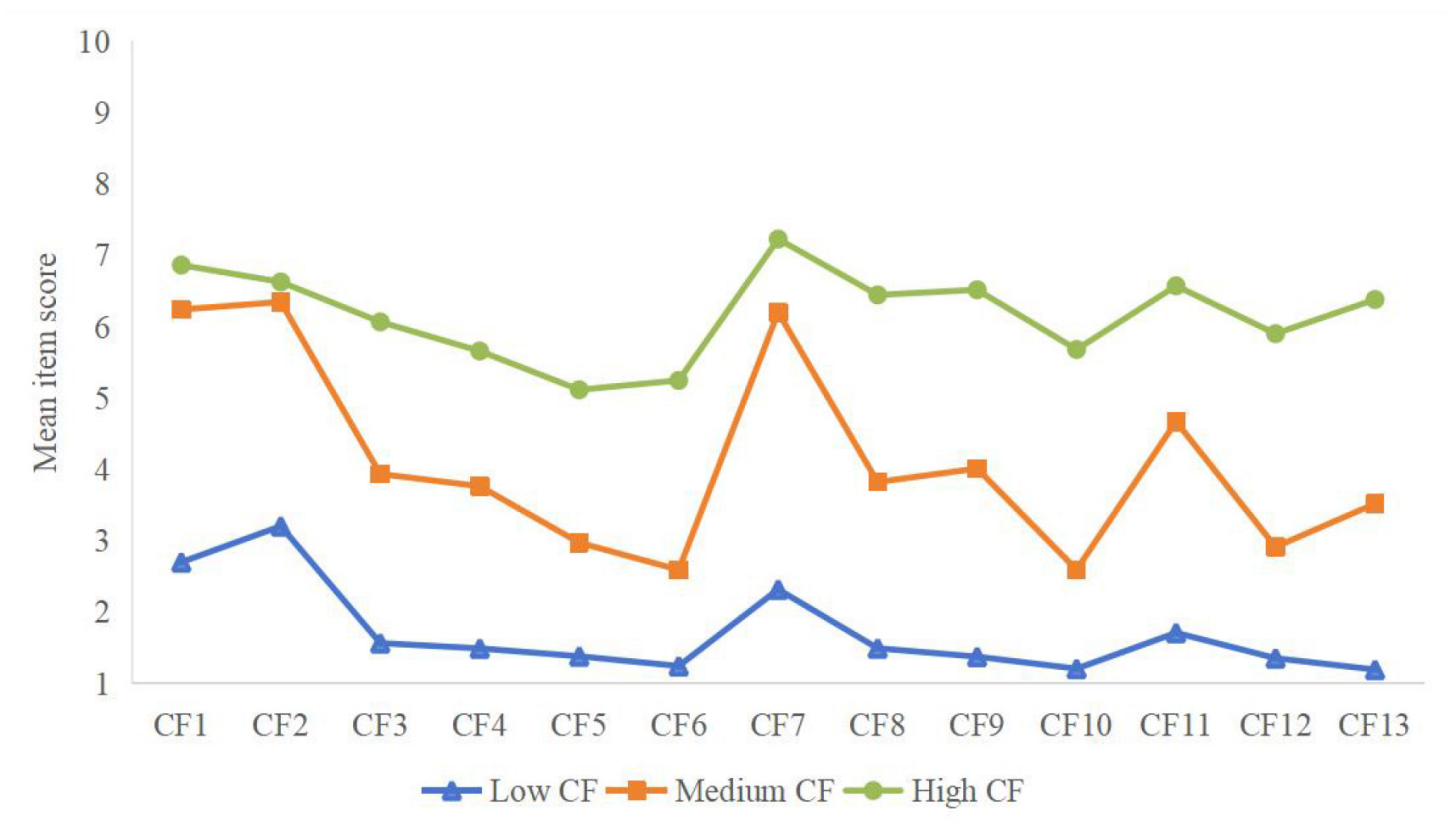
Distribution of scores of three potential characteristics of nurses' CF.

According to the Compassion Fatigue Scale, the severity of CF can be categorized into low (1–4), moderate (4–7), and high (7–10) levels based on the mean item score. Higher mean item scores indicate more severe CF. This scale's categorization criteria can provide a reference for the delineation of three potential profiles. As shown in Table 2 and Figure 1, Category 1 was the most numerous, accounting for 44.8% (203/453). Scores for secondary trauma and burnout, encompassing CF, were lower than those for Categories 2 and 3. As shown in Figure 1, the mean scores for the 13 items ranged from 1 to 4. This suggests that these nurses possessed effective coping mechanisms and support systems in the face of work stress and emotional burden. Therefore, this category was designated “Low CF.” Category 2 was the second most numerous, accounting for 34.2% (115/453). Scores for secondary trauma and burnout, encompassing CF, were intermediate between those for Categories 1 and 3. As shown in Figure 1, the mean scores for the 13 items ranged from 4 to 7. This suggests that these nurses may be experiencing some degree of CF, but not yet reaching high levels, remaining at an average level. Therefore, it is designated “Medium CF.” Category 3 was the smallest, accounting for 21% (95/453). CF, encompassing secondary trauma and burnout, had higher scores than categories 1 and 2. As shown in Figure 1, the average scores for 13 items were roughly in the 7–10 range. This suggests that these nurses experience significant CF in their work, facing significant emotional exhaustion and burnout. Therefore, it is designated “High CF.”

### 3.3 Relationship between nurses' resilience and CF

Pearson correlation analysis showed that resilience was negatively correlated with nurses' CF (r = −0.422, *P* < 0.01). For details, see Table 3.

**Table 3:** Correlation between nurse' resilience and CF.

Variables	Tenacity	Strength	Optimism	Resilience	Secondary traumatic stress	Burnout	CF
Tenacity	1						
Strength	0.906^**^	1					
Optimism	0.816^**^	0.815^**^	1				
Resilience	0.981^**^	0.960^**^	0.884^**^	1			
Secondary traumatic stress	−0.329^**^	−0.382^**^	−0.318^**^	−0.359^**^	1		
Burnout	−0.398^**^	−0.448^**^	−0.383^**^	−0.429^**^	0.817^**^	1	
CF	−0.390^**^	−0.443^**^	−0.375^**^	−0.422^**^	0.926^**^	0.974^**^	1

^**^ indicates *P* < 0.01.

### 3.4 Characteristics of latent profile membership

Table 4 shows the comparison of demographic characteristics and work-related factors among the three latent profiles of nurses' CF. Chi-square tests showed statistically significant differences among the groups in monthly income, number of patients cared for daily, number of night shifts per week, and health status (*P* < 0.05). To compare the differences in resilience among the three latent profiles of nurses' CF, we conducted a one-way analysis of variance. As shown in Table 5, resilience and its three dimensions showed significant differences among the three latent profiles of nurses' CF (*P* < 0.05). Because resilience and its three dimensions were normally distributed and met the homogeneity of variance test, we performed *post hoc* pairwise comparisons using the SNK q test. The SNK q test results showed that resilience scores were highest in the Low CF group, while resilience scores were lowest in the High CF group.

**Table 4:** Differences in latent characteristics of CF among nurses in terms of demographics (*N* = 453).

Variables	Total	Low CF (*n* = 203)	Medium CF (*n* = 155)	High CF (*n* = 95)	χ2	*P*
		*n* (%)	*n* (%)	*n* (%)		
Gender					1.075	0.584
Male	47 (10.4)	18 (8.9)	17 (11)	12 (12.6)		
Female	406 (89.6)	185 (91.1)	138 (89)	83 (87.4)		
Age (years)					7.600	0.269
≤ 25	84 (18.5)	43 (21.2)	28 (18.1)	13 (13.7)		
26–30	131 (28.9)	59 (29.1)	47 (30.3)	25 (26.3)		
31–40	215 (47.5)	87 (42.9)	76 (49)	52 (54.7)		
>40	23 (5.1)	14 (6.9)	4 (2.6)	5 (5.3)		
Education level					7.403	0.116
College and below	57 (12.6)	28 (13.8)	14 (9)	15 (15.8)		
Undergraduate	346 (76.4)	159 (78.3)	117 (75.5)	70 (73.7)		
Postgraduate	50 (11)	16 (7.9)	24 (15.5)	10 (10.5)		
Marital status					3.221	0.200
Married	258 (57)	125 (61.6)	82 (52.9)	51 (53.7)		
Unmarried/divorced/widowed	195 (43)	78 (38.4)	73 (47.1)	44 (46.3)		
Working years					1.839	0.765
1–5	177 (39.1)	82 (40.4)	63 (40.6)	32 (33.7)		
6–10	106 (23.4)	48 (23.6)	33 (21.3)	25 (26.3)		
>10	170 (37.5)	73 (36)	59 (38.1)	38 (40)		
Labor relationship					2.256	0.324
Contractor	386 (85.2)	175 (86.2)	127 (81.9)	84 (88.4)		
Employee	67 (14.8)	28 (13.8)	28 (18.1)	11 (11.6)		
Monthly income (CNY)					11.730	0.019
< 5,000	87 (19.2)	51 (25.1)	20 (12.9)	16 (16.8)		
5,000–10,000	345 (76.2)	140 (69)	128 (82.6)	77 (81.1)		
>10,000	21 (4.6)	12 (5.9)	7 (4.5)	2 (2.1)		
Number of daily nursing patients					14.509	0.006
1–5	222 (49)	111 (54.7)	77 (49.7)	34 (35.8)		
6–10	154 (34)	65 (32)	55 (35.5)	34 (35.8)		
>10	77 (17)	27 (13.3)	23 (14.8)	27 (28.4)		
Number of night shifts per week					11.769	0.019
≤ 1	220 (48.6)	113 (55.7)	70 (45.2)	37 (38.9)		
2	172 (38)	65 (32)	68 (43.9)	39 (41.1)		
3	61 (13.5)	25 (12.3)	17 (11)	19 (20)		
Health status					24.006	< 0.001
Health	182 (40.2)	106 (52.2)	47 (30.3)	29 (30.5)		
Sub-health	242 (53.4)	90 (44.3)	94 (60.6)	58 (61.1)		
Suffering from disease	29 (6.4)	7 (3.4)	14 (9)	8 (8.4)		

Bold indicates statistically significant variables; χ2, Chi-square test.

**Table 5:** Comparison of resilience scores among the three latent profiles of CF (*N* = 453).

Variables	Total sample (*N* = 453) M ±SD	Low CF (*N* = 203) M ±SD	Medium CF (*N* = 115) M ±SD	High CF (*N* = 95) M ±SD	F	*P*	SNK q
Tenacity	33.91 ± 8.34	37.46 ± 8.00	31.79 ± 7.02	29.77 ± 8.02	41.441	< 0.001	1 > 2 > 3
Strength	22.04 ± 5.03	24.45 ± 4.61	20.77 ± 4.18	18.95 ± 4.72	57.984	< 0.001	1 > 2 > 3
Optimism	10.16 ± 2.74	11.27 ± 2.64	9.45 ± 2.41	8.96 ± 2.61	35.599	< 0.001	1 > 2 > 3
Resilience	66.11 ± 15.43	73.18 ± 14.46	62.01 ± 12.80	57.67 ± 14.82	49.867	< 0.001	1 > 2 > 3

M, Mean; SD, standard deviation; F, One-way ANOVA; SNK q, Student-Newman-Keuls q test.

### 3.5 Predictors of latent profile membership

The latent profile of nurses‘ CF was used as the dependent variable, High CF was used as the reference group, and the variables with statistically significant differences in Tables 4, 5 (monthly income, number of patients cared for daily, number of daily nursing patients, health status, and resilience) were used as independent variables for multivariate logistic regression analysis. The results showed that the number of daily nursing patients, number of night shifts per week, and resilience were influencing factors of the latent profile of nurses' CF (*P* < 0.05), as shown in Table 6.

**Table 6:** Multivariate logistic regression analysis of latent profiles of CF in nurses (*N* = 453).

Variables	B	SE	Wald χ2	P	OR	95% CI
Low CF vs. High CF						
Resilience	0.080	0.011	51.598	**< 0.001**	1.084	(1.06, 1.108)
Monthly income (refer: >10,000 CNY)						
< 5,000CNY	−0.141	0.895	0.025	0.875	0.869	(0.15, 5.023)
5,000–10,000CNY	−0.533	0.846	0.396	0.529	0.587	(0.112, 3.082)
Number of daily nursing patients (refer: >10)						
1–5	1.422	0.389	13.332	**< 0.001**	4.146	(1.932, 8.894)
6–10	0.760	0.394	3.729	0.053	2.138	(0.989, 4.625)
Number of night shifts per week (refer: 3)						
≤ 1	0.539	0.416	1.677	0.195	1.714	(0.758, 3.877)
2	0.287	0.418	0.471	0.493	1.332	(0.587, 3.024)
Health status (refer: suffering from disease)						
Health	0.425	0.619	0.471	0.493	1.530	(0.454, 5.151)
Sub-health	0.182	0.592	0.094	0.759	1.199	(0.376, 3.825)
Medium CF vs. High CF						
Resilience	0.025	0.011	5.856	**0.016**	1.026	(1.005, 1.047)
Monthly income (refer: > 10,000 CNY)						
< 5,000 CNY	−0.993	0.899	1.218	0.270	0.371	(0.064, 2.16)
5,000–10,000 CNY	−0.569	0.839	0.460	0.498	0.566	(0.109, 2.932)
Number of daily nursing patients (refer: >10)						
1–5	1.244	0.370	11.288	**0.001**	3.468	(1.679, 7.165)
6–10	0.697	0.369	3.566	0.059	2.007	(0.974, 4.135)
Number of night shifts per week (refer: 3)						
≤ 1	0.879	0.416	4.463	**0.035**	2.409	(1.066, 5.445)
2	0.880	0.409	4.639	**0.031**	2.412	(1.082, 5.373)
Health status (refer: suffering from disease)						
Health	−0.464	0.538	0.744	0.388	0.628	(0.219, 1.805)
Sub-health	−0.216	0.496	0.190	0.663	0.806	(0.305, 2.13)

Bold indicates statistically significant variables, Refer, Reference group.

Specifically, nurses with higher resilience were more likely to be classified as Low and Medium CF. Nurses who cared for 1–5 patients on a daily basis were more likely to be assigned to Low and Medium CF than nurses who cared for >10 patients on a daily basis. In addition, nurses who worked ≤ 2 night shifts per week were more likely to be classified as Medium CF than nurses who worked 3 night shifts per week.

## 4 Discussion

### 4.1 Latent profile characteristics of CF in nurses

This study aims to analyze the differences in nurses‘ CF through latent profiles. The results of the study divided nurses' CF into three latent profiles based on the scores of the 13 items included in CF, namely “Low CF,” “Medium CF” and “High CF,” which proved that our research hypothesis 1 was established. The results of this study showed that among the three latent profiles of CF, Low CF accounted for the largest proportion (44.8%), followed by Medium CF (34.2%) and High CF (21.0%). This is similar to the results of previous studies. Yi et al. (61) and Yin et al. (52) conducted LPA on the CF of nursing interns and ICU nurses, respectively, and also found that CF can be divided into three latent profiles, with Low CF and Medium CF accounting for the largest proportion. This classification reveals that there is heterogeneity in CF among different nurse groups. It not only enriches and expands the previous overall research perspective on nurses‘ CF, but also provides a reference for formulating relevant intervention measures to alleviate nurses' CF.

The number of people with low CF was the largest, accounting for 44.8% (203/453). The proportion of this group was higher than that of the study by Fu et al. (62), which may be related to the fact that the research group of Fu et al. (62) was operating room nurses. The total score of CF in this group was 22.11 ± 7.47, the secondary traumatic stress score was 6.99 ± 2.73, and the occupational burnout score was 15.13 ± 5.70. This shows that both the total score of CF and the scores of the two dimensions were lower than medium CF and high CF. This shows that the nurses in this group were able to effectively maintain emotional stability and professional commitment when facing work pressure and emotional burden. It is worth noting that this group scored the highest in the CF2 item (“I think I have not successfully achieved my life goals”), which is consistent with the research results of Yi et al. (61). This shows that although this type of nurses performed well in terms of emotional burden, they may be dissatisfied with the realization of their personal goals, suggesting that attention should be paid to the nurses‘ professional identity and personal value realization. Research has shown that nurses' CF is a dynamic process that begins with the compassion experience phase and worsens over time, ultimately developing into the CF phase (63). Therefore, regular assessment of nurses‘ CF is crucial. For such nurses, professional development training can be provided as early as possible, or outstanding nurses' contributions can be recognized to enhance their sense of professional pride. This will help prevent their CF from worsening.

The proportion of medium CF was 34.2% (115/453), which shows that some nurses still have a medium level of CF, which is similar to the results of Yi et al. (61). The total score of CF in this group was 53.45 ± 9.39, the secondary traumatic stress score was 16.16 ± 5.31, and the burnout score was 37.29 ± 7.84. This shows that although these nurses experienced moderate emotional exhaustion at work, they still had certain coping mechanisms. In this group, the CF1 item (“I feel that my work bothers me”) and the CF2 item (“I don't think I have successfully achieved my life goals”) scored higher, indicating that nursing work has brought a certain degree of emotional distress to these nurses. Therefore, nurses can relieve stress through self-care. For example, talk to relatives or colleagues and seek help, actively participate in group activities or insist on physical exercise (64). These methods can help reduce the stress and negative emotions caused by work (27).

High CF accounted for 21.0% (95/453), the smallest proportion of nurses, which is similar to the results of previous studies (52). The CF score of this group was 80.38 ± 13.24, the secondary traumatic stress score was 29.24 ± 6.36, and the burnout score was 51.14 ± 9.19. This indicates that this group of nurses is in a severe CF stage. It is worth noting that among the three latent categories of CF, the burnout dimension scores were significantly higher than secondary traumatic stress. This finding indicates that when nurses face high-intensity work pressure and emotional challenges, burnout may be their more prominent psychological reaction. In particular, the CF7 item in CF (description of feeling weak or exhausted after work) scored the highest, indicating that nurses generally have a sense of burnout from nursing work (65). Therefore, this type of nurses should pay special attention to their physical health, actively adjust their work mood, develop and cultivate their personal hobbies, and it is also very important to reasonably divide the boundaries between work and life (31). At the same time, hospital management should provide nurses with necessary work resources, pay attention to their mental health, develop targeted CF resilience training programs (66, 67), and implement mindfulness-based professional resilience interventions (68). These measures will help reduce nurses' occupational stress, reduce emotional exhaustion, and improve their overall wellbeing.

### 4.2 Influencing factors of the latent profile of nurses' CF

Our results showed that resilience, the number of patients cared for daily, and the number of night shifts per week may be influencing factors of the latent profile of nurses' CF, which confirmed Hypotheses 2 and 3.

First, nurses with higher levels of resilience were more likely to be classified as Low CF and Medium CF. This suggests that resilience may be an important factor influencing CF, which is consistent with previous research findings (40, 44). Nurses with higher levels of resilience generally have stronger psychological adaptability and emotional regulation abilities. Even when faced with various difficulties and challenges at work, they are able to use various resources around them to overcome their negative emotions (62). In addition, they regularly provide psychological counseling to themselves to maintain their physical and mental health. This self-care not only helps improve the quality of patient care but also contributes to their career development (69). Therefore, medical managers should develop appropriate intervention measures to improve nurses‘ resilience and regard it as an important protective trait against CF (70). Medical managers can regularly hold resilience training courses to teach nurses emotional regulation and stress management skills, including cognitive behavioral therapy or mindfulness meditation (71). Through cognitive behavioral therapy, nurses will learn to identify negative thoughts and use effective emotion regulation strategies to cope with occupational stress, thereby enhancing their psychological strength (72). This psychological strength enables nurses to remain resilient in the face of challenges and reduce the occurrence of CF. At the same time, the practice of mindfulness meditation will encourage nurses to pay attention to their present experiences and enhance self-awareness. This practice is helpful in reducing nurses' burnout, improving their sleep quality and increasing their resilience (73). In addition, these training courses should also provide practical opportunities to encourage nurses to apply the skills they have learned in their daily work. Through group discussions or the establishment of WeChat groups, nurses can share each other's experiences and challenges, build a strong peer support network, and enhance collective resilience. Finally, hospital managers can establish a mechanism for regular assessment of nurses‘ mental health status and understand nurses' needs and challenges through questionnaires and interviews. Help nurses identify potential mental health problems, provide professional psychological counseling services, ensure that nurses can receive timely psychological support, and further enhance their ability to resist CF.

Secondly, compared with nurses who care for more than 10 patients on a daily basis, nurses who care for 1–5 patients on a daily basis are more likely to be assigned to Low CF and Medium CF. This suggests that the number of patients cared for may be an influencing factor in nurses‘ CF, which is similar to previous research results (74, 75). This may be because when nurses care for fewer patients, they can better understand the specific needs of each patient and establish a closer doctor-patient relationship. This will help improve nurses' job satisfaction and reduce the risk of emotional exhaustion. However, when nurses need to care for too many patients, the workload will increase significantly, making it difficult for them to devote enough time and energy to understand the specific situation of each patient. This situation may lead to a decline in nursing quality, lack of energy, and weakened compassion, and will also aggravate nurses‘ burnout and CF (74, 75). Medical managers can effectively address this problem by reasonably allocating work demands and resources (31). First, managers should evaluate the existing nurse-patient ratio and flexibly adjust the nurses' workload according to the patient's condition and nursing needs. Second, implement a hierarchical nursing system, divide patients into different levels according to their nursing needs, and ensure that the number of patients each nurse is responsible for matches their nursing capabilities. An appropriate nurse-patient ratio can not only reduce the burden on nurses but also improve patient safety and the quality of nursing services (76).

Finally, nurses who work ≤ 2 night shifts per week are more likely to be classified as Medium CF than those who work 3 night shifts per week. This suggests that the number of night shifts per week may be an influencing factor for nurses‘ CF, which is similar to previous research results (62, 77). For nurses who work more night shifts per week, frequent night shifts can disrupt their normal biological clock and lead to decreased sleep quality (62). Studies have shown that night shift workers are more likely to suffer from insomnia, fatigue, and inattention, which directly affect the severity of nurses' CF (78). Relatively speaking, nurses who work no more than 2 night shifts per week face fewer challenges. This arrangement allows nurses to maintain a more regular work and rest schedule and enhance their body's recovery ability. Good sleep and adequate rest can help improve concentration and work efficiency, thereby reducing fatigue at work. In addition, fewer night shifts also allow nurses to have more time to maintain family and social relationships, enhance life satisfaction and professional pride, and this good quality of life can in turn promote their mental health. Therefore, it is recommended that nursing managers can optimize the scheduling system. For example, the frequency of night shifts should be reduced, and nurses should be limited to no more than two night shifts per week (78), giving nurses more time to work during the day shift. Secondly, a reasonable shift system should be implemented to allow nurses to have better time rotation, reduce the continuity of night shift work, and allow nurses to have an adaptation period between night and day shifts. Through these optimizations, the working environment of nurses can be effectively improved, and their mental health and job satisfaction can be enhanced.

## 5 Enlightenment on innovation of nursing practice

This study, examining the latent profiles of nurses‘ CF, not only reveals the emotional challenges they face but also provides important insights for innovation in nursing practice. First, the results suggest that nurses' CF can be categorized into three latent profiles: low, medium, and high. For nurses with low CF, managers should encourage them to maintain emotional stability and professional engagement in their work. Although these nurses perform well in terms of emotional burden, they still need to focus on achieving their personal goals. For nurses with medium and high CF, managers should provide emotional regulation and stress management training tailored to their specific needs. Because these nurses face higher levels of emotional distress, helping them develop effective coping mechanisms is crucial. These differences in emotional burden across different groups suggest that interventions should consider individual characteristics to more specifically address their needs.

Our research also found that resilience may be a significant factor influencing nurses' CF. Management can regularly conduct resilience training and enhance nurses' mental health through a variety of courses and activities. For example, using methods such as cognitive behavioral therapy or mindfulness meditation can not only strengthen nurses' resilience but also improve their ability to cope with workplace stress. Hospitals should establish ongoing mental health support mechanisms and regularly assess nurses' mental wellbeing and work demands. Providing professional psychological counseling services and support networks to ensure nurses receive timely help when needed will effectively reduce the incidence of CF. Furthermore, balancing the number of patients under care is crucial for reducing CF. Management should regularly assess the nurse-patient ratio to ensure that nurses can fully understand and address the needs of each patient while maintaining a reasonable workload. Establishing close doctor-patient relationships will enhance nurses' job satisfaction and reduce the risk of emotional exhaustion. The impact of night shift work on nurses' mental health should also not be ignored. Optimizing scheduling should minimize the number of night shifts per week. This arrangement can reduce the disruption of night shifts to the circadian rhythm, improve sleep quality, and thus enhance nurses' focus and work efficiency. Managers may also consider implementing flexible shift systems that allow nurses an adjustment period between day and night shifts, thereby enhancing their physical and psychological recovery.

In fact, CF among nurses is widespread worldwide. Although this study focused on Chinese nurses, its findings also have implications for nursing practice in other countries. By implementing the measures outlined above, nursing practice can become more humane and sustainable.

## 6 Limitations

Our study also has some limitations. (1) This study only selected nurses from six hospitals in Sichuan Province, China for investigation. The interpretation of the research results was conducted in the context of Chinese culture and failed to represent the situation of nurses in other regions. The geographical limitation of the sample may affect the generalizability of the results. Future studies can conduct stratified sampling across regions or countries to improve the reliability and extrapolation of the results. (2) This study adopted a cross-sectional design and cannot determine the causal relationship between resilience and CF. Future studies can consider a longitudinal research design, based on a latent variable mixed growth model, to track the trajectory of the latent profile of CF in different nurse groups over time. In order to more comprehensively understand the dynamic relationship between these variables. (3) This study only conducted the survey through online electronic questionnaires, which may have self-report bias. The participants' answers may be affected by social expectations, thus affecting the authenticity of the results. Therefore, more objective data can be collected in the future to improve the authenticity of the research results. (4) This study mainly explored the impact of resilience and demographic variables on the latent profile of CF in nurses, and has not yet explored other possible influencing factors, such as social support and emotional intelligence. Future research could consider incorporating these variables or further exploring the heterogeneity of CF and its influencing factors in different work settings (e.g., emergency department, ICU, and operating room). This would help to more fully understand the complexity of CF in nurses and provide stronger support for improving nursing practice.

## 7 Conclusion

Our study breaks through previous studies on nurses' CF and explores the heterogeneity of nurses' CF from an innovative perspective. Through LPA, we identified three latent profiles of nurses' CF, low CF, medium CF, and high CF. We found that resilience, the number of patients cared for daily, and the number of night shifts per week may be important factors affecting the latent profile of CF. To this end, we recommend that medical managers take the following measures: (1) provide nurses with resilience interventions based on cognitive behavioral therapy or mindfulness therapy; (2) reasonably adjust the nurse-patient ratio according to the patient's condition and implement a graded nursing system; (3) optimize the scheduling system and try to limit nurses to no more than two night shifts per week. Rationally implement a shift system to reduce the continuity of night shift work. Our research results suggest that hospital management should pay attention to the mental health and workload of nurses, which provides a reference for nursing managers to develop personalized intervention measures for nurses with different characteristics.

## Data Availability

The raw data supporting the conclusions of this article will be made available by the authors, without undue reservation.
